# Formation and loss of metastable brucite: does Fe(II)-bearing brucite support microbial activity in serpentinizing ecosystems?

**DOI:** 10.1098/rsta.2018.0423

**Published:** 2020-01-06

**Authors:** A. S. Templeton, E. T. Ellison

**Affiliations:** Department of Geological Sciences, University of Colorado, Boulder, CO 80309-0399, USA

**Keywords:** brucite, microbe, electron, metastable, hydrogen

## Abstract

Ultramafic rocks undergo successive stages of hydration and oxidation during water/rock interaction, giving rise to secondary minerals such as brucite, serpentine, magnetite and the production of H_2(g)_. Ferroan brucite $\left(\text{M}{\text{g}}_{\text{x}}{\text{Fe}}_{\left(1-\text{x}\right)}^{2+}{\left(\text{OH}\right)}_{2}\right)$ often forms under low water/rock ratios early during the ‘serpentinization’ process. The formation of ferroan brucite sequesters Fe(II) and suppresses the production of H_2_, thereby limiting the flux of reductants suitable for sustaining microbial metabolism. Yet ferroan brucite is a relatively soluble mineral ‘reservoir’ for reactive Fe(II). Brucite is often metastable and can be lost at later stages of peridotite hydration when there is a significant increase in the water/rock ratio or the activity of SiO_2_ or CO_2_. The Fe(OH)_2_ component of brucite has the thermodynamic potential to reduce most aqueous oxidants. Therefore, ferroan brucite may reduce water and/or dissolved carbon, nitrogen and sulfur species, while the Fe(II) is converted into more stable secondary minerals such as Fe(II/III)-oxides and hydroxides (e.g. green-rust, magnetite, iowaite and pyroaurite) and ferric serpentine. The reactivity of ferroan brucite, and the associated rate of Fe solubilization and oxidation in subsurface fluids, could be a key regulator on the rate of electron transfer from serpentinites to the rock-hosted biosphere. Aqueous alteration of ferroan brucite may significantly modulate the H_2_ activity in fluids circulating within partially serpentinized rocks, and buffer H_2_ as it is lost by advection or *in situ* consumption by a hydrogenotrophic microbial community. Moreover, there may be microbial organisms that specifically colonize and use ferroan brucite as an electron donor for their metabolism. The energy fluxes sustained by localized brucite oxidation may often be sufficiently large to sustain abundant microbial communities; water/rock reaction zones where brucite is consumed could serve as environments to search for extant or fossil serpentinite-hosted life.

This article is part of a discussion meeting issue ‘Serpentinite in the Earth System’.

## 1. Introduction

Rocks composed of olivine and pyroxene spontaneously react with water at near-surface temperatures and pressures. ‘Serpentinization’, the hydration and oxidation of ultramafic rocks, commonly results in the formation of serpentine minerals, a diversity of Fe(II/III) hydroxides and oxides, and hydrogen gas (e.g. equation (1.1)). The rate and extent of serpentinization, Fe-oxidation and associated H_2_ production is highly dependent on several environmental factors, such as temperature, modal composition of the rock, water/rock ratio, and whether or not the system is open or closed to fluid advection. Since H_2_ is such a potent reductant that can be used in biological metabolism, serpentinization reactions and associated H_2_ production are integral to investigations regarding how water/rock reactions can support chemosynthetic life.

1.1 $$\begin{array}{l} \underset{\mathrm{olivine}}{{\text{Mg}}_{1.8}{\text{Fe}}_{0.2}{\text{SiO}}_{4}}+\underset{\text{serpentine }}{{\text{H}}_{2}\text{O}\to {\left(\text{Mg, Fe}\right)}_{\text{3}}\text{S}{\text{i}}_{2}{\text{O}}_{\text{5}}{\left(\text{OH}\right)}_{\text{4}}}+\underset{\text{ brucite }}{(\text{Mg, Fe}){\left(\text{OH}\right)}_{2}}+\underset{\text{magnetite}}{\text{F}{\text{e}}_{\text{3}}{\text{O}}_{\text{4}}+{\text{H}}_{2}}. \end{array}$$

This review paper will focus on the importance of brucite $\left(\text{M}{\text{g}}_{\text{x}}{\text{Fe}}_{\left(1-\text{x}\right)}^{2+}{\left(\text{OH}\right)}_{2}\right)$ as a metastable secondary mineral, and will present the hypothesis that brucite formation and loss may be integral to the habitability of serpentinites. Peridotite hydration is well known to be a multistage process, and brucite is a product and a reactant during serpentinization, due to changing stability as water/rock reaction progresses. We highlight that brucite is also a reactive Fe(II) reservoir that may play a critical role in regulating the flux of H_2_ generated during successive stages of rock alteration. The metastability of Fe(II)-bearing brucite creates a conundrum for predicting habitability based upon energy availability, because brucite can ‘throttle’ the release of reducing equivalents from the rock system. When the formation of ferroan brucite suppresses H_2_ generation, this should limit the availability of bioavailable energy sources. When Fe(II)-brucite is subsequently oxidized, it may give rise to high rates of hydrogen generation that may be used by microbial life.

We briefly review the geochemical processes that may control the formation and loss of ferroan brucite through reaction with fluids. We then explore how brucite may regulate the extent of hydrogen generation during progressive water/rock reaction. Since any geochemical or biological process that can destabilize ferroan brucite may significantly change the habitability of low-temperature serpentinizing systems, we suggest that it is time to develop a mechanistic understanding of how brucite reactivity creates conditions that sustain rock-hosted life.

## 2. Review

### (a) Brucite $\left(\text{M}{\text{g}}_{\text{x}}{\text{Fe}}_{\left(1-\text{x}\right)}^{2+}{\left(\text{OH}\right)}_{2}\right)$ formation during serpentinization

Brucite $\left(\text{M}{\text{g}}_{\text{x}}{\text{Fe}}_{\left(1-\text{x}\right)}^{2+}{\left(\text{OH}\right)}_{2}\right)$ is a common phase in partially serpentinized rocks. Brucite typically forms during the earliest stages of serpentinization (e.g. [1]), and is often localized around olivine [2] or is intimately intermixed within serpentine veins. Brucite is typically most abundant under low water/rock conditions, where the activities of the FeO, MgO and SiO_2_ components of the system are controlled by the primary peridotite mineral assemblage [3]. Brucite forms because olivine contains stoichiometrically insufficient SiO_2_ for complete conversion to serpentine. Thus, brucite tends to be more prevalent in olivine-rich rocks that hydrate under low silica activity, such as dunites (e.g. [4,5]), and brucite can comprise 5–20% of the secondary mineral assemblage.

While reaction of pyroxene minerals can set the SiO_2_ activity higher during peridotite hydration, and cause serpentine to form instead of brucite, olivine/brucite equilibria do develop in harzburgites due to strong gradients in silica activity between pyroxenes and olivine [6]. Brucite can also accumulate in harzburgites undergoing water/rock interaction at low temperatures, since olivine alters more rapidly than pyroxene, giving rise to fluids buffered to lower silica activity [4].

Metastable brucite can also form even when serpentine or talc are thermodynamically favoured at high silica activity. Nesbitt & Bricker [7] discuss the relative rates of olivine and orthopyroxene dissolution, compared to the formation of secondary mineral products during serpentinization. They infer that the precipitation of the hydrous alteration phases is slower than the rate of primary mineral dissolution, such that fluids may almost reach saturation with olivine. Brucite, the most soluble secondary mineral, will then accumulate as a metastable phase and persist until it is converted to serpentine (a reaction which itself can be kinetically sluggish, see §2b).

**Figure 1.**
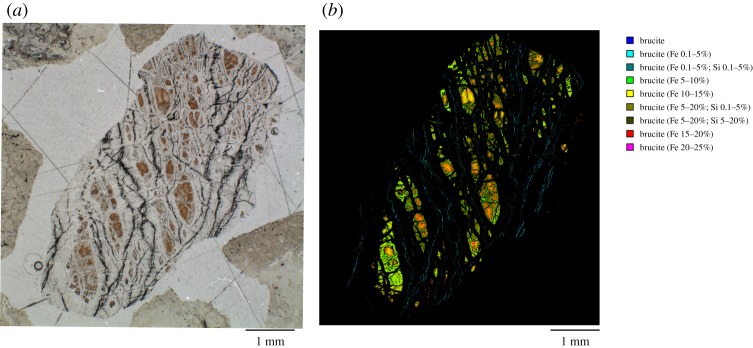
(*a*) Optical image of rock cuttings recovered during rotary drilling at 70 m depth in the Oman Water Ministry well NSHQ14, with opaque magnetite in vein centres, a colourless serpentine matrix, and orange-brown areas dominated by oxidized Fe-brucite. (*b*) chemical image showing the range of Fe incorporation into the brucite solid solution compositions observed in this sample, defined as nine different brucite compositions based on the amount of Si and Fe. (Online version in colour.)

**Figure 2.**
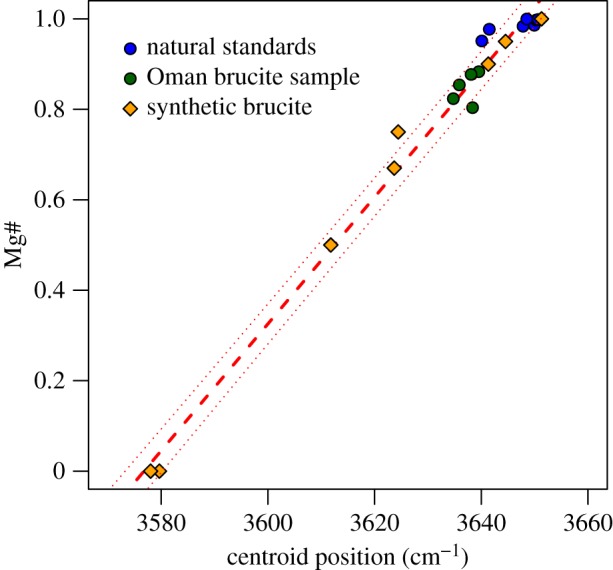
Raman spectroscopic determination of Fe content of brucite, expressed as Mg#, from the centroid position of the *ν*_1_ O-H stretch Raman peak. Calibration is based on 20 natural and synthetic brucite samples. Dotted lines represent the 1*σ* prediction interval for Mg# (±0.05). (Online version in colour.)

A significant portion of the Fe in partially serpentinized rocks resides in brucite [3,5,6,8–11]. The Mg# of brucite (where Mg# = Mg/(Mg + Fe) mole ratio) can often vary from 0.95 to much lower values such as 0.65 when there is a notable Fe(OH)_2_ component [2,12,13] (figure 1). Anomalously ferroan brucite, with Mg#s as low as 0.4 have also been observed in highly reduced mineral assemblages in serpentinite [14]. Strongly ferroan brucite, particularly the Fe(OH)_2_ endmember, can mineralogically be considered as ‘amakinite’, (Fe^II^,Mg)(OH)_2_, as classified by the International Mineralogical Association. See box 1 for details on detecting and quantifying Fe in brucite-amakinite using EDS mapping (figure 1) and Raman spectroscopy (figure 2).

Box 1. **Detecting and quantifying Fe in brucite.**

Brucite is often fine-grained and intimately intergrown with serpentine (e.g. [1,13–15]), which can render it both difficult to detect and quantify in partially serpentinized rocks. Brucite can be detected at the bulk scale using X-ray Diffraction (XRD). At the microscale, electron-microprobe analysis (EMPA) of polished thin sections can be used to identify zones of brucite mineralization, or in brucite–serpentine mixtures. For example, low SiO_2_ contents in EMPA or energy dispersive spectroscopy (EDS) mineralogy mapping of serpentine can be used to determine the presence of brucite and the overall Fe content. However, it can be challenging to partition Fe between the coexisting serpentine and brucite in any pixel, and many analyses may be required in order to extrapolate the Fe content of the serpentine and brucite endmembers. Automated mineralogy platforms based on quantitative EDS provide another approach to detecting Fe-bearing brucite. For example, in figure 1, we show the variable Fe(II) substitution into brucite in partially serpentinized peridotite recovered from 70 m depth in the subsurface during rotary drilling in the Northern Sharqiyah region in the Samail ophiolite in Oman. These drill chips were obtained in 2004 by the Ministry of Regional Municipalities, Environment and Water Resources of Oman. This sample has been previously characterized by Miller *et al*. [13] by X-ray diffraction, EMPA and Raman spectroscopy. The optical mineralogy shows opaque magnetite in vein centres, colourless serpentine matrix, and orange-brown areas dominated by Fe-bearing brucite. Quantitative EDS spectral acquisition, matrix corrections and peak deconvolutions were conducted using the Mineralogic® (Zeiss) automated mineralogy system to quantitatively show the range of $\text{M}{\text{g}}_{\text{x}}{\text{Fe}}_{\left(1-\text{x}\right)}^{2+}{\left(\text{OH}\right)}_{2}$ solid solution compositions that can be found in a single sample at a 1.5 micron pixel resolution. Each pixel is categorized with a series of mineral definitions imposed on the measured elemental composition. This sample shows nine different brucite compositions based on the amount of Si and Fe, and only the brucite has been coloured in the image shown (i.e. only pixels with low silicon less than 5 wt%, except for some pixels with 5–20 wt% Fe and 5–20 wt% Si). Brucite pixels have been coloured according to their iron composition, with Fe-rich pixels highlighted by warmer colours and generally concentrated in the core centres of the mesh texture. Fe-poor brucite is observed associated with magnetite in the vein centres.

Raman imaging is another quantitative characterization approach that can be used for rapid, non-destructive microscale point-analysis or mapping of brucite distribution and Fe concentration. The *ν*_1_ O-H stretch Raman peak (3652 cm^−1^ for Mg(OH)_2_) is sensitive to Fe substitution in the brucite structure, shifting to lower wavenumbers until it reaches 3576 cm^−1^ in Fe(OH)_2_, the amakinite end-member. We have developed a calibration for the Fe^2+^ content of brucite based on the position of this peak using a suite of laboratory-synthesized and natural mineral brucite standards (figure 2). The calibration was performed by isolating and baseline-subtracting the brucite O-H stretch peak. The centroid of the peak was calculated as $\nu_{\text{c}}=\left(\sum \nu i\right)/\sum \nu$ where is *ν*_c_ is the centroid position, *ν* is the Raman shift in the measured spectrum and *i* is the measured Raman intensity at *ν*. Seven brucite specimens purchased from mineral dealers (Mg# 0.95–1 determined by EPMA), five points from a serpentinized Oman peridotite sample (Mg# 0.82–0.88 determined by EPMA), and eight samples synthesized in the laboratory by coprecipitation (Mg# 0–1) were used in the calibration. Mg# was predicted by the linear formula $\text{Mg}\#=0.014\nu_{c}-50.15$, with a 1*σ* prediction interval of ±0.05. Since Raman spectroscopy probes molecular bond structure, this technique is sensitive only to Fe substituted into the brucite structure, and is unaffected by intimately intergrown phases such as serpentine, which may also contain Fe. Since serpentine polymorphs are robustly identified by the O-H stretch Raman peaks in the same spectral region [16], this information can be collected simultaneously in a spectrum or hyperspectral map.

Thermodynamic modelling has shown that the extent of Fe(II) incorporation in brucite should be highly temperature dependent. McCollom & Bach [17] estimated the thermodynamic properties of Fe-bearing brucite, and predicted that there is a strong preferential partitioning of Fe(II) into $\text{M}{\text{g}}_{\text{x}}{\text{Fe}}_{\left(1-\text{x}\right)}^{2+}{\left(\text{OH}\right)}_{2}$, particularly at temperatures below 200°C. When ferroan brucite forms, it locks Fe(II) in the secondary mineral structure and prevents hydrogen generation [6,18]. Therefore, McCollom & Bach [17] predict the hydrogen activity will precipitously decline as a function of temperature, due to the thermodynamic equilibrium between brucite, magnetite and H_2_ (equation (2.1)).

2.1 $$\text{3Fe}{\left(\text{OH}\right)}_{2}↔\text{F}{\text{e}}_{\text{3}}{\text{O}}_{\text{4}}\text{ + }{\text{H}}_{2}\text{ + 2}{\text{H}}_{2}\text{O}\text{.}$$

The Fe-composition of brucite can strongly modulate the effective H_2_ concentration in serpentinites [19–22]. Therefore, environmental controls on the partitioning of Fe(II) into brucite are important for predicting the extent of hydrogen production during serpentinization. The importance of brucite-amakinite as a reservoir of Fe(II) should be particularly pronounced in systems undergoing low-temperature serpentinization, which can often encompass large volumes of fractured rock and fluids circulating within the known temperature limits for life-activity (e.g. ≤122°C [23]).

### (b) Brucite reactivity and implications for H_2_ generation

Brucite (Mg_x_Fe_(1−x)_(OH)_2_) formed during early stages of water/rock interaction is later consumed through several possible pathways. Often brucite is no longer stable as serpentinization proceeds. For example, Beard *et al*. [2] report the formation of early serpentine and ferroan brucite mineral assemblages during the initial alteration of olivine-rich rocks from the Atlantis Massif (Hole 1309D). These authors infer that magnetite then forms at the expense of brucite when the activity of SiO_2_ is sufficiently high to give rise to the instability of ferroan brucite. Similarly, Bach *et al*. [5] first observe the replacement of olivine rims, and then cores, by serpentine and ferroan brucite in Site 1274 along the Mid-Atlantic Ridge. These authors then invoke a second stage of reaction at high silica activities, resulting in the formation of late-stage magnetite and serpentine produced from the breakdown of ferroan brucite. Silica involved in the consumption of brucite can be derived from other minerals such as plagioclase [2] or orthopyroxene [5]. Observations from peridotite rocks undergoing modern low-temperature serpentinization have also indicated that H_2_ generation may in part be modulated by ferroan brucite reacting with SiO_2(aq)_. Miller *et al*. [24] analysed the mineralogy and Fe-speciation of partially serpentinized rocks in Oman as a function of depth, and identified rocks at depth where ferroan brucite was intimately intergrown with serpentine. However, the serpentinites closer to the surface were brucite-poor, and contained more magnetite and ferric serpentine. They proposed a coupled Fe-oxidation and silicification reaction, where brucite conversion to magnetite and Fe(III)-rich serpentine gives rise to low-temperature H_2_ production.

The destabilization of ferroan brucite by aqueous silica can form Fe-rich serpentine without magnetite. Hydrogen can be evolved if the mineral conversion involves the oxidation of Fe(II) and incorporation of Fe(III) into serpentine. The reaction below (equation (2.2)), derived from Klein *et al*. [6], represents the oxidation and remineralization of the Fe(OH)_2_ component in brucite into ferric serpentine through reaction with dissolved silica.

2.2 $$\text{2Fe}{\left(\text{OH}\right)}_{2}\text{ + 2Si}{\text{O}}_{\text{2( aq) }}\text{ + }{\text{H}}_{2}\text{O = F}{\text{e}}_{2}\text{S}{\text{i}}_{2}{\text{O}}_{\text{5}}{\left(\text{OH}\right)}_{\text{4}}\text{ + }{\text{H}}_{2}\text{.}$$

In other instances, the Fe(OH)_2_ component of brucite is partially oxidized and mineralized as magnetite (e.g. equation (2.1)), also yielding H_2_ with magnesian serpentine and/or brucite as a product (e.g. [3]).

However, fluid–mineral equilibrium is often not achieved in natural systems, and brucite can persist in the presence of fluids with high SiO_2(aq)_ concentrations [25,26]. For example, metastable olivine/brucite equilibrium has been maintained for at least short durations in high temperature closed-system experiments, in high SiO_2(aq)_ fluids where serpentine should instead be stable [26]. Recent open-system experiments by Tutolo *et al*. [27] demonstrate that at 150°C, the rates of olivine dissolution, brucite dissolution and brucite precipitation are all faster than the rate of brucite reaction with SiO_2(aq)_ to form thermodynamically favoured serpentine. Silica tetrahedra must polymerize onto brucite sheets to form silicates, such as serpentine and talc, and this process can be the rate-determining step [28]. The rate of the silicification reaction can be highly sensitive to SiO_2(aq)_, and thus sensitive to the extent of SiO_2(aq)_ delivery (i.e. flow paths introducing dissolved silica) and accessibility of mineral surfaces to advecting fluid (e.g. some brucite surfaces quickly react but then prevent further interaction between silica-bearing fluid and residual brucite) [27]. Thus brucite metastably persists in many high SiO_2(aq)_ systems, including the Fe(II) stored within its structure. However, despite some of the physical and kinetic barriers to converting brucite to serpentine, most rocks that have undergone full serpentinization only contain minor residual brucite [2], so brucite conversion reactions do ultimately go to completion.

**Figure 3.**
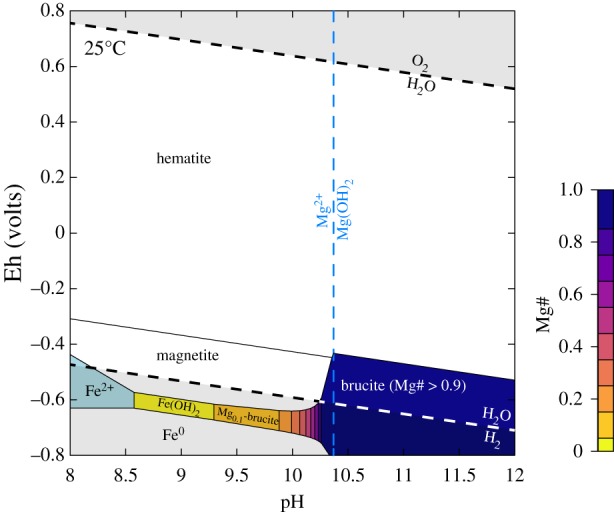
Eh–pH diagram at 25°C and 1 atm predicting the stability of brucite (Mg_1−x_Fe_x_(OH)_2_) variably substituted with Fe^2+^ in equilibrium with a solution containing 5 × 10^–5^ M Fe^2+^(aq) and Mg^2+^(aq). The $\Delta G_{f}^{\circ }$ of Fe-brucite (Mg_1−x_Fe_x_(OH)_2_) was calculated based on ideal solid solution behaviour between Fe(OH)_2_ and Mg(OH)_2_ (see text). Vertical dashed line indicates the pH of saturation with respect to magnesian brucite (Mg(OH)_2_) for 5 × 10^−5^ M Mg^2+^(aq). (Online version in colour.)

The Fe(OH)_2_ component in brucite is a strong reductant, and because of brucite's relatively fast dissolution kinetics and high reactivity, it can impose reducing conditions on the system as a whole if it is present. The conversion of brucite to magnetite (with Fe(III)/Fe_T_ ∼0.66) is often considered to buffer the *f*H_2_, as shown previously in equation (2.1). The thermodynamic properties of the Fe(OH)_2_ end-member have been estimated by numerous authors, while the thermodynamic properties of the ferroan brucite solid solutions found in nature are quite unconstrained other than through assumptions of ideal behaviour (e.g. [17]). In a Fe Pourbaix diagram we constructed at surface temperatures and pressures (e.g. 25°C, 1 atm) using $\Delta G_{f}^{\circ }$ of Fe(OH)_2_ from McCollom & Bach [17], $\Delta G_{f}^{\circ }$ of other species from the SUPCRT92 thermodynamic database [29], and $\Delta G_{f}^{\circ }$ of Fe-brucite calculated based on ideal solid solution behaviour between Fe(OH)_2_ and Mg(OH)_2_, brucite phases containing more than 10% Fe(OH)_2_ in the solid solution should poise a system below the lower stability limit of water and thus at high H_2_ fugacity (figure 3).

However, there are several other ferrous-ferric hydroxides, with Fe(III)/Fe_T_ closer to 0.33, that may instead form first from Fe(OH)_2_ oxidation, affecting the equilibrium *f*H_2_. For example, Fe-dominated ‘Green Rust’ group minerals can form when anions are present to balance excess charge in their double-layered hydroxide structure [30–32]. Anions such as chloride, bromide, sulfate, carbonate and hydroxide can play an important role in Fe(OH)_2_ oxidation, and fill the interlayer for charge compensation in layered hydroxides with variable Mg : Fe ratios and Fe(III)/Fe_T_ (e.g. [30,33]). Recently, Tosca *et al*. [34] experimentally traced Fe(II) reactivity in alkaline, anoxic solutions. As they increased pH above pH 8 and precipitated Fe(OH)_2_, they observed water reduction, giving rise to concurrent production of reduced species (inferred to be H_2_) and green rust formation. The green rust then continued to convert into magnetite through further ‘ageing’.

Fe(II/III)-serpentine, iowaite or pyroaurite are other potential products of brucite oxidation reactions. The exact nature of the secondary mineral product derived from ferroan brucite oxidation should be strongly determined by the environmental conditions, such as the hydrogen fugacity, the activity of SiO_2_, the availability of CO_2_ and pH. The mechanistic steps involved in brucite transformation will strongly modulate the kinetics of Fe(II) oxidation and hydrogen generation.

When the Fe(OH)_2_ component in brucite becomes fully oxidized, iowaite (Mg_6_Fe^(III)^_2_(OH)_16_Cl_2_.4H_2_O) can be produced, with the uptake of Cl^−^ to balance Fe(III) in the structure [35]. Since iowaite is dominated by Fe(III), iowaite has often been inferred to form under relatively oxidizing conditions generated by the infiltration of seawater (e.g. in sites such as the Izu-Bonin forearc, the Iberian margin and the Mid-Atlantic Ridge [35–37]).

Brucite dissolution and carbonation in the presence of elevated CO_2_ has been intensively studied as a form of CO_2_ sequestration, since brucite is a metastable secondary phase that can then react with CO_2_ at moderate temperatures (e.g. [38]). Experimental reaction with CO_2_ has shown the replacement of brucite by amorphous Mg-carbonate phases, such as nesquehonite, as well as hydroxycarbonates (e.g. [39]); the potential formation of stable MgCO_3_ is of interest for carbon sequestration [40,41]. Boschi *et al.* [12] show that the presence of brucite in serpentinites plays a notable role in the natural carbonation process, particularly in dunite lithologies that are brucite-rich.

However, the chemical behaviour of the Fe(OH)_2_ component is not always investigated. When ferroan brucite reacts with CO_2_, Boschi *et al*. [12] report that the brucite is replaced with Fe-Mg layered double hydroxides in the pyroaurite-coalingite solid solution of Mg^2+^-Fe^3+^ carbonates. Similarly, Hostetler *et al*. [1] invoke the conversion of ferroan brucite to pyroaurite-coalingite phases during reaction with CO_2_ in groundwaters. Clearly at least some of the Fe(II) stored in brucite is oxidized during carbonation reactions. While CO_2_ may be accompanied by O_2_ in atmospherically equilibrated fluids, which could easily oxidize the Fe(OH)_2_ component, in anoxic fluids it is also possible that the reducing equivalents are transferred to other redox active species, such as nitrate, nitrite or even CO_2_, giving rise to reduced N and C species as well as H_2_ production.

The potential direct reduction of CO_2_ by brucite has not been studied, although brucite could function as a labile energy source and potent reductant for CO_2_ reduction. For example, ferroan brucite may serve to initiate the formation of metastable carbon species essential to prebiotic synthesis of organic compounds. In geological systems, CO_2_ reduction by H_2_ is predicted to occur under highly reducing conditions when appropriate mineral catalysts are present, such as magnetite, Fe and Fe-Ni sulfides, or Fe-Ni alloys such as awaruite [42–45]. We suggest that when brucite is intimately associated with these phases, this may provide a co-localization of mineral-derived reducing power and catalysis.

Holm *et al*. [46] invoke ferroan brucite and its transformation to magnetite as a potential foci for organic synthesis reactions. In particular, Holm *et al*. [46] note that brucite is an important mineral for scavenging phosphate and borate from fluids, thereby localizing these components to catalytic surfaces of layered hydroxides and magnetite under reducing conditions favourable for the abiotic formation of organic compounds. One class of brucite-borate reactions of particular interest is the potential alkaline formation of ribose, the pentose sugar that plays several key roles in biochemistry. A class of important brucite-phosphate reactions is the potential formation of polyphosphate, which may have promoted phosphorylation reactions central to early biochemistry [46]. Mechanistic studies of brucite-driven reduction or polymerization of any C, N or P will be required to substantiate any of these ideas. However, we do reiterate that ferroan brucite may be able to power important carbon reduction reactions. For example, Barge *et al*. [47] recently demonstrated that Fe(OH)_2_ could drive the reduction of pyruvate to lactate; when Fe(II/III) bearing green rusts were present, pyruvate and ammonium could react to form the amino acid alanine.

### (c) Connections between brucite and microbial metabolism

Ferroan brucite is rarely discussed as a substrate for microbial metabolism, despite the fact that it is such a potent reductant. Fe(II) in brucite may serve as an electron donor when coupled with oxygen, nitrate or other powerful electron acceptors, similar to microbial Fe(II)-oxidation of basalts or olivine (e.g. [48–50]). Microbial Fe(II)-oxidation could be particularly prevalent in moderately alkaline $\text{Mg}\text{-}\text{HC}{{\text{O}}_{\text{3}}}^{-}$ fluids (i.e. ‘type I’ fluids *sensu* Barnes & O'Neil [51]) in serpentinizing systems, where more oxidizing fluids would react with ferroan brucite, and the Fe(II) is predicted to be fully converted to Fe(III) in smectites or ferric hydroxides such as goethite or pyroaurite, depending upon the environmental conditions.

Jöns *et al*. [52] do assess Fe(OH)_2_ as a catabolic substrate and they identify the large amount of free energy potentially available from converting Fe(OH)_2_ to ferric hydroxide by organisms coupling Fe(II)-oxidation to O_2_ reduction (e.g. equation (2.3)).

2.3 $$4\text{Fe}{\left(\text{OH}\right)}_{2}+{\text{O}}_{2(\text{aq})}↔4\text{Fe}(\text{OOH})+2{\text{H}}_{2}\text{O}\text{.}$$

We note that ferroan brucite is a sufficiently strong reductant (figure 3) that it can also be coupled to much less powerful oxidants, including CO_2_ and sulphate, depending upon *in situ* activities of the relevant species. For example, microbial methanogenesis or sulphate reduction may be feasible with the Fe(OH)_2_ component of brucite as the reductant

2.4 $$\text{12Fe}{\left(\text{OH}\right)}_{2}+\text{ S}{{\text{O}}_{\text{4}}}^{2-}+{\text{H}}^{+}↔\text{4F}{\text{e}}_{\text{3}}{\text{O}}_{\text{4}}+\text{H}{\text{S}}^{-}+\text{ 12}{\text{H}}_{2}\text{O }$$

and

2.5 $$12\text{Fe}{\left(\text{OH}\right)}_{2}+\text{C}{\text{O}}_{2}↔4\text{F}{\text{e}}_{3}{\text{O}}_{4}+\text{C}{\text{H}}_{4}+10{\text{H}}_{2}\text{O}\text{.}$$

Therefore, brucite might serve as a biological energy source under highly reducing conditions as well.

It is not known whether the Fe(II) in brucite could be directly oxidized biologically, or whether molecular hydrogen must be produced as an intermediate. In the hypothetical scenarios in equations (2.4) and (2.5), the Fe(OH)_2_ component of brucite is shown as the direct electron donor for sulphate and CO_2_ reduction. Extracellular electron transfer between microbial organisms and minerals or electrodes is an increasingly recognized pathway for minerals to serve as electron donors or acceptors in microbial energy generation (see review by Shi *et al*. [53] and examples therein). Biological electron uptake from solids is also commonly invoked in microbial corrosion of metallic surfaces. Dinh *et al*. [54] observed rapid, direct electron transfer between Fe^0^ and surface-attached *Desulfobacterium* sp. sulphate reducers, as well as between Fe^0^ and *Methanobacterium* sp. methanogens. There are also several types of microbial ‘hydrogenotrophs’ that can use solid cathodes poised at low redox potential. For example, several methanogens have been shown to promote H_2_ generation at carbon cathodes [55], with and without redox mediators such as methyl viologen. Some methanogens can also use solid electrodes poised at potentials below −500 mV to reduce CO_2_ to methane or acetate (e.g. [56,57]), without significant H_2_ production and consumption as an intermediate.

In both metal corrosion and electrode-based studies, it is not yet clear whether electron transfer occurs directly between the solid surface and an attached cell. Recent studies by Deutzmann *et al*. [58] have questioned whether methanogens are mediating direct electron transfer between redox active surfaces and the cell, or whether they facilitate the production of H_2_ or formate as an intermediate electron carrier. Microbial hydrogenases are ubiquitous metalloenzymes that can reversibly catalyse the production and consumption of H_2_, and Deutzmann *et al*. [58] demonstrated that extracellular hydrogenases excreted by methanogens and acetogens can adsorb to electrode surfaces and produce H_2_. Thus they show that free hydrogenase enzymes that have been released from cells can stably catalyse the formation of electron donors that are then rapidly consumed by nearby cells.

**Figure 4.**
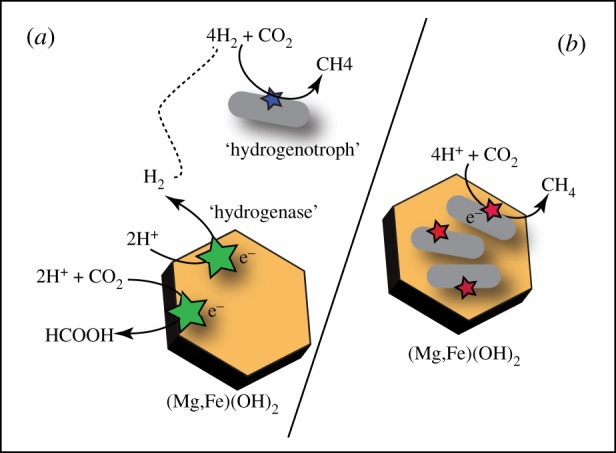
Conceptual diagram of Fe-substituted brucite serving as a hypothetical electron donor for microbial metabolism. (*a*) ‘Indirect’ pathway of transferring reducing equivalents from mineral (brucite) to microbe (hydrogenotroph) mediated by extracellular hydrogenases sorbed to the brucite surface. We depict these hydrogenases attached to brucite mediating proton reduction to H_2_, or potentially direct CO_2_ reduction to C1-compounds such as formate. (*b*) Direct electron transfer between an attached microbial surface and structural Fe(II) in brucite, to be used for example for CO_2_-reduction metabolisms such as methanogenesis (or acetogenesis or other nominally hydrogenotrophic metabolisms). (Online version in colour.)

Typically, it is assumed that hydrogen is first produced from Fe-brucite oxidation as shown in equation (2.1) or equation (2.2), and is then used directly in ‘hydrogenotrophic’ metabolism. For example, Schulte *et al*. [59] invoked Fe(OH)_2_ oxidation to produce magnetite, water and H_2_ (equation (2.1)) as a source of H_2_ for chemolithotrophs and biochemical processes such as methanogenesis. In these scenarios, microorganisms take up H_2_ produced during water/rock reactions using hydrogenases. In serpentinizing systems, molecular studies have identified a diversity of Fe-Ni and Fe-Fe hydrogenases [60,61]. In addition, ‘hydrogenotrophs’ such as methanogens, acetogens and sulphate reducers are common. Hydrogenotrophic methanogens such as *Methanobacterium* sp. have been identified in the fluids circulating through the Del Puerto ophiolite in Northern California [62], the Zambales ophiolite in the Philippines [63], the Samail Ophiolite in Oman [64], and the Cedars in California [65]. It is assumed that these methanogens consume H_2(aq)_ produced through prior water/rock interaction. However, it is possible that some of these H_2_-consuming organisms can colonize redox active minerals poised at low potentials, such as Fe-bearing brucite (Mg_x_Fe_(1−x)_(OH)_2_), and mediate direct electron transfer. Similarly, it is possible that extracellular hydrogenases can promote Fe(II)-oxidation and hydrogen production from ferroan brucite (see concept depicted in figure 4).

### (d) When and where might brucite reactivity generate habitable conditions?

In seeking habitable conditions on Earth and on other rocky bodies undergoing water/rock interaction in our solar system, we often seek energy sources and large chemical disequilibria that can be sustained for sufficiently long time scales to enable life to flourish. The strongly reducing, H_2_-rich hyperalkaline fluids common in serpentinizing systems are generally not habitable on their own, given the strong limitation imposed by scarce carbon and substrates that can serve as oxidants. Therefore, one of the guiding strategies for seeking life in serpentinizing systems is to identify fluid mixing zones, such as where H_2_-rich hydrothermal fluids are discharged from ultramafic rocks into lower pH, more oxidizing fluids. Excellent modern examples include locales such as Lost City hydrothermal field near the Mid-Atlantic Ridge, the Prony Bay Hydrothermal Field, New Caledonia, and springs in Samail Ophiolite, Oman and the Cedars, California, to name just a few locales [66–71]. It has also been proposed to seek chemical disequilibria in the subsurface, either where moderately alkaline and oxidizing fluids mix with upwelling hyperalkaline reduced fluids (e.g. shallower $\text{Mg-HC}{{\text{O}}_{3}}^{-}$ ‘type I’ fluids mixing with deeper Ca-OH ‘type II’ fluids, *sensu* Barnes & O'Neil [51]), or at contact zones between crust and mantle rocks, such as zones where gabbro and peridotite are juxtaposed [64]. Such contact zones between fluids with notable differences in SiO_2(aq)_ activity also tend to possess key differences in the availability of oxidants/reductants.

During peridotite hydration, a change in fluid regime from low to higher water rock ratio, or from more closed to open-system conditions, can dramatically change the stability and abundance of brucite and Fe(II). Studies of modern and fossil systems note how a change to open-system conditions, with advection of fluids with low H_2_, or with fluids with high silica activities (as two discrete examples), may destabilize brucite and form more thermodynamically favoured serpentine and magnetite phases. The high SiO_2(aq)_ can be due to the sluggish precipitation of serpentine and talc, due to the late stage alteration of pyroxene once olivine is almost fully consumed, or be generated by water/rock reaction with adjacent or cross-cutting gabbros or pyroxenites, giving rise to silica metasomatism (e.g. [4,36,72]). Boschi *et al*. [73,74] and Seyfried *et al*. [25] have investigated fluid exchange between gabbro rocks and peridotites at Lost City, and invoke silica metasomatism as the mechanism destabilizing brucite from the alteration assemblage. Similarly, Tutolo *et al*. [27] model how advective fluxes of SiO_2(aq)_ drive partial brucite conversion to serpentine.

Brucite dissolution rates are highly sensitive to pH (e.g. [28]), and therefore also susceptible to a change from closed- to open-system conditions. The influx of lower pH fluids from gabbros into peridotite can contribute to the brucite loss near fault zones. More commonly, when an increase in water/rock ratio is accompanied by a change in fluid chemistry, such as the introduction of a less-reacted, lower pH fluid, brucite dissolution can lead to large voids, as well as generate a source of labile Fe(II)_(aq)_ to participate in the redox reactions described previously. Jöns *et al.* [52] traced the formation of cavities in serpentinized harzburgites, demonstrating how brucite formed under static conditions dissolves and form voids lined with new Fe-rich precipitates when there is an incursion of new fluids that are far from equilibrium with respect to brucite.

Therefore, the timing and localization of any change to open system conditions will fundamentally affect the stability of ferroan brucite, and exert a major control on the rate and extent of H_2_ production, which in turn affects predictions of the habitability and availability of bioavailable energy sources. Several recent microbiological studies have all invoked potential H_2_ production or ‘buffering’ from brucite oxidation as a potentially key pathway for sustaining populations of microbial ‘hydrogenotrophs’ such as methanogens, acetogens and sulphate reducers [24,52,64,75,76]. It raises the tantalizing possibility that microbial activity could be preferentially localized to subsurface interfaces where $\text{M}{\text{g}}_{\text{x}}{\text{Fe}}_{\left(1-\text{x}\right)}^{2+}{\left(\text{OH}\right)}_{2}$ loss is actively occurring. Serpentinite core samples that contain interfaces where ferroan brucite is reacting out have not yet been intensively studied for evidence of biomass accumulation or life activity, although such tests will be feasible with partially-serpentinized dunites and harzburgites recently obtained through the Oman Drilling Project (http://omandrilling.ac.uk).

Recent lipid biomarker studies have successfully identified preserved evidence of microbial activity in late-stage vein mineralization in systems where brucite loss and remineralization has occurred. Klein *et al*. [77] detected large accumulations of organic matter preserved in late-stage magnesian brucite. They specifically identified accumulations of lipids from microbial colonies in carbonate–brucite chimneys that formed from the mixing of peridotite-derived hydrothermal fluids with seawater at the Iberian Margin. Zwicker *et al*. [76] also detected lipid biomarkers within carbonate–brucite veins formed where fluid mixing had occurred at the Chimaera site in Turkey. They infer that the hydromagnesite/Mg-brucite assemblage has formed from the replacement of ferroan brucite by CO_2_-bearing solutions. These authors also invoke Fe-brucite oxidation as a potential energy source for archaeal methanogenesis and bacterial sulphate reduction.

## 3. Perspective: future efforts to quantify the role of ferroan brucite in sustaining microbial activity in low-temperature serpentinizing systems

To assess the habitability of a serpentinizing system, it is imperative to evaluate the spectrum of reductants derived from water/rock interaction, their fluxes through time, and their potential to mix and favourably react with oxidants. In low temperature water/rock systems, it is often assumed that the rate and extent of hydrogen production could be severely limited, due to the slow rate of olivine hydrolysis. In addition, at temperatures below 150°C, Fe(II) will be increasingly trapped into brucite (e.g. [17]), and pyroxene alteration will also be sluggish, helping to maintain low SiO_2(aq)_ activities (e.g. [36]). These predictions can lead to the assumption that in low-temperature serpentinizing systems, the minimum energy requirements for microbial life cannot be met. However, if metastable brucite is transitory, ferroan brucite can buffer the H_2_ activity of partially serpentinized rocks. Importantly, the rate of H_2_ production will vary through time.

If any of the ‘triggers’ for brucite transformation occur, particularly the change from closed system to open system conditions, decrease in pH or the addition of reactive ligands, this could be followed by a massive increase in H_2_ production rate. In addition, it is possible that *in situ* biological activity, particularly the local consumption of H_2_, could be a driver for further destabilizing brucite and pulling the oxidation reactions forward (i.e. buffering effect). Thus one of the overarching goals of this review is to articulate that low-temperature serpentinizing systems should evolve to a state where brucite loss will become a dominant feature of the system, and associated Fe(II) release and oxidation may generate H_2_ at a notably rapid rate, thereby creating habitable conditions if sufficient oxidants and chemical disequilibria are present.

However, we cannot yet model the temporal evolution of brucite-modulated H_2_ generation and loss in serpentinites. The kinetics of key reactions, such as the brucite silicification reaction, are not constrained across enough of the relevant serpentinization conditions, as discussed by Tutolo *et al*. [27]. Moreover, there are many other unknowns regarding brucite reactivity. For example, Schrauzer & Guth [78] show a strong pH dependence of Fe(OH)_2_ driven production of H_2_, with a maximum near pH 9. Their data implies that there is a complex rate law that may be dependent upon both Fe(OH)_2_ solubility and Fe oxidation. These authors also invoke Fe(OH)_2_ disproportionation (forming Fe^3+^ and metallic Fe^0^) as a key step in brucite reaction sequences, which has mechanistic and kinetic implications if true. Experimental studies will be required to further validate whether phases such as metallic iron (Fe^0^) can be a reaction intermediate in the production of H_2_. In addition, the Schrauzer & Guth [78] experiments suggest H_2_ production can be strongly suppressed if reducing equivalents from Fe(OH)_2_ are diverted to N or C reduction reactions. The probability of Fe(OH)_2_ driving CO_2_, nitrate, nitrite or N_2_ reduction under environmental conditions has not been sufficiently studied, and therefore it is not yet possible to predict how the aqueous geochemistry may modulate the dominant reduction reactions during ferroan brucite oxidation. Similarly, the effect of consumptive loss of H_2_, through advective fluid flow or microbial activity, cannot yet be modelled due to lack of kinetic data on how the mineral–water equilibria would respond.

If microbial organisms can preferentially solubilize brucite to release Fe(II) for reaction, or mediate direct electron transfer as hypothesized in figure 4, life activity could significantly affect the reaction progress and secondary mineralization in serpentinites. As Schulte *et al*. [59] note, when there is a large possible change in free energy, yet the approach to equilibrium is slow, then there is an opportunity for biological catalysis. Now that some of the dominant methanogens from serpentinites have been cultured, such as the *Methanobacterium* sp. NSHQ4 investigated by Miller *et al*. [79], it will be tractable to conduct experiments examining the reactivity of $\text{M}{\text{g}}_{\text{x}}{\text{Fe}}_{\left(1-\text{x}\right)}^{2+}{\left(\text{OH}\right)}_{2}$ in the presence of purified extracellular hydrogenases or when in direct cell contact. More broadly, we recommend testing whether or not organisms can directly or indirectly accelerate the conversion of $\text{M}{\text{g}}_{\text{x}}{\text{Fe}}_{\left(1-\text{x}\right)}^{2+}{\left(\text{OH}\right)}_{2}$ to a diversity of Fe(II/III)-oxide and silicate products. This would be possible by directly incubating hydrogenotrophic organisms isolated from serpentinizing systems with Fe(II)-substituted brucite in anoxic aqueous solutions. It will be important to explore the role of biological H_2_ or electron utilization on the rate of brucite oxidation, which could be determined by comparison of the extent of Fe(III) production in sterile versus biologically active experiments. The extent of direct cell/mineral contact could also be assessed. It is possible that brucite can only temporarily serve as a cathode, if electron-transfer rates decrease as a function of mineral oxidation. Would the potential rates of electron uptake or hydrogenase activity be sufficient to maintain metabolism, or would the reaction become self-limiting as brucite is transformed into mixed valence mineral products?

To best probe the physiology of organisms that may directly use brucite as an energy source, we suggest targeted environmental culturing of anaerobic organisms adapted to using ferroan brucite as a substrate. Natural Fe-bearing brucite can be purchased through mineral vendors, or synthesized with variable Fe(OH)_2_ content through coprecipitation of ferrous iron and magnesian chloride salts with base under highly reducing conditions. Brucite could then be deployed in field-based mineral/water incubations, such as in downhole experiments placed in drill holes in ophiolite systems or in ultramafic massifs on the seafloor. The environmentally colonized brucite could be studied in the laboratory to assess the microbe/mineral interactions, and used to enrich and isolate hydrogenotrophic methanogens, acetogens or sulphate reducers using minimal medium amended with CO_2_ or sulphate but no external source of H_2_. Such strategies might be particularly useful to identify organisms that can oxidize Fe(II) in brucite directly through novel surface-associated electron transfer mechanisms.

At the systems scale, it is also now possible to probe brucite-rich rocks recovered from the subsurface in systems undergoing modern water/rock interaction, and test whether or not there is evidence of notable life activity or biomass accumulation and preservation at reaction interfaces where there is extensive loss of Fe(II)-brucite. Recent drilling in the Oman ophiolite (Oman Drilling Project, http://omandrilling.ac.uk) and at the Atlantis Massif (IODP expedition 357, http://www.ecord.org/expedition357/) are examples of international projects that have provided new access to excellent sample sets for these investigations. It is time to determine whether there is a greater abundance, diversity or specific functionality of microbial organisms that can establish themselves where ferroan brucite is the key regulator for energy transfer from the rock to the biological system.

## 4. Conclusion

When ferroan brucite accumulates during serpentinization, as it is especially prone to do during low temperature water/rock interaction, it represents stored reducing potential that can be released by subsequent geochemical reactions. Ferroan brucite is likely to become destabilized by a change to open system conditions, including advection of fluids that may be less alkaline, more oxidizing, or contain SiO_2_ or CO_2_. The addition of these components, coupled with the release of reducing power from brucite, could produce habitable conditions, and a variety of microbial metabolisms may be fuelled by reducing equivalents from ferroan brucite. However, the key mechanisms and rates governing the release of this reducing power, and how it might be harnessed by microorganisms, remain important topics for ongoing research.

## Supplementary Material

Supplementary Data 1

## Supplementary Material

Supplementary Data 2

## Data accessibility

Raw data used to generate figure 1 and figure 2 can be accessed in .csv and .tiff format through a electronic supplementary materials.
