# Supplementary figures and images for: Formation and loss of metastable brucite: does Fe(II)-bearing brucite support microbial activity in serpentinizing ecosystems?
Source: Philos Trans A Math Phys Eng Sci. 2020 Jan 6;378(2165):20180423. doi: 10.1098/rsta.2018.0423 (PMC7015309; doi:10.1098/rsta.2018.0423)

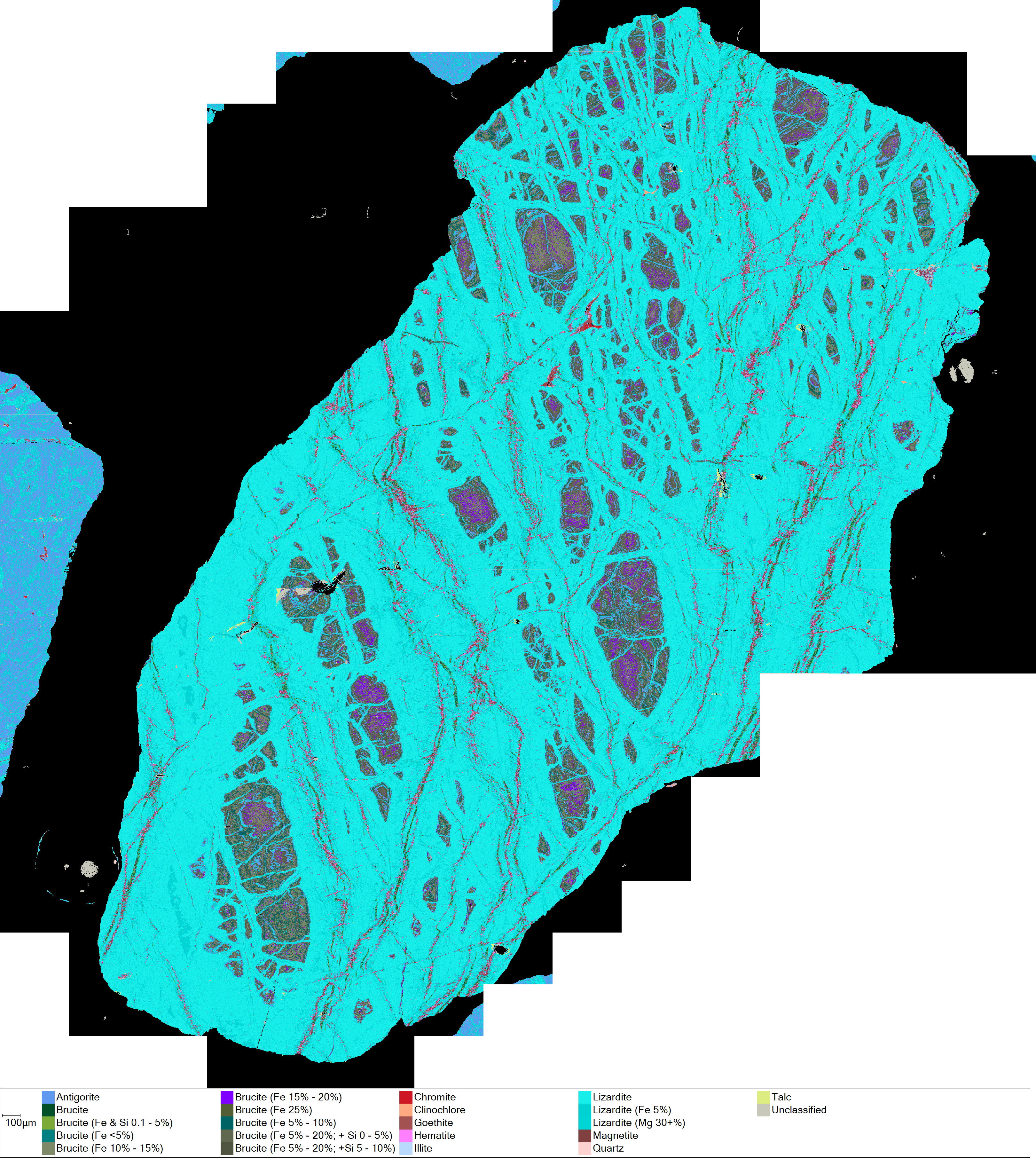

Supplement: Supplementary Data 1 [file rsta20180423supp1.tiff]
